# Variability in synovial inflammation in rheumatoid arthritis investigated by microarray technology

**DOI:** 10.1186/ar1903

**Published:** 2006-02-16

**Authors:** Johan Lindberg, Erik af Klint, Ann-Kristin Ulfgren, André Stark, Tove Andersson, Peter Nilsson, Lars Klareskog, Joakim Lundeberg

**Affiliations:** 1 Department of Biotechnology, AlbaNova University Center, Royal Institute of Technology, S-106 91 Stockholm, Sweden; 2 Department of Rheumatology, Karolinska Institutet, Karolinska University Hospital, 171 76 Stockholm, Sweden; 3 Department of Orthopedics, Karolinska University Hospital, 171 76 Stockholm, Sweden

## Abstract

In recent years microarray technology has been used increasingly to acquire knowledge about the pathogenic processes involved in rheumatoid arthritis. The present study investigated variations in gene expression in synovial tissues within and between patients with rheumatoid arthritis. This was done by applying microarray technology on multiple synovial biopsies obtained from the same knee joints. In this way the relative levels of intra-patient and inter-patient variation could be assessed. The biopsies were obtained from 13 different patients: 7 by orthopedic surgery and 6 by rheumatic arthroscopy. The data show that levels of heterogeneity varied substantially between the biopsies, because the number of genes found to be differentially expressed between pairs of biopsies from the same knee ranged from 6 to 2,133. Both arthroscopic and orthopedic biopsies were examined, allowing us to compare the two sampling methods. We found that the average number of differentially expressed genes between biopsies from the same patient was about three times larger in orthopedic than in arthroscopic biopsies. Using a parallel analysis of the tissues by immunohistochemistry, we also identified orthopedic biopsies that were unsuitable for gene expression analysis of synovial inflammation due to sampling of non-inflamed parts of the tissue. Removing these biopsies reduced the average number of differentially expressed genes between the orthopedic biopsies from 455 to 171, in comparison with 143 for the arthroscopic biopsies. Hierarchical clustering analysis showed that the remaining orthopedic and arthroscopic biopsies had gene expression signatures that were unique for each patient, apparently reflecting patient variation rather than tissue heterogeneity. Subsets of genes found to vary between biopsies were investigated for overrepresentation of biological processes by using gene ontology. This revealed representative 'themes' likely to vary between synovial biopsies affected by inflammatory disease.

## Introduction

Rheumatoid arthritis (RA) is a common chronic inflammatory disease, so far defined by a set of criteria [1] rather than by a knowledge of the underlying molecular pathogenesis. Substantial efforts have been made to characterize the synovial inflammation in RA, and during these studies it has become evident that there is a large variability in cell content and in protein expression, both within single joints and between patients with RA [2-7]. This variation also exists at the gene expression level [8]. Microarray (MA) technology allows the expression of thousands of genes to be monitored simultaneously and can thus increase the understanding of the complicated molecular processes of joint inflammation in more detail than has been possible with immunohistochemistry and related techniques [9-14]. Recently reviewed [15], MA has been used to acquire knowledge about RA in various experimental systems with the use of both cell cultures [16-22] and biopsies [23-30] obtained from the synovium. So far, MA has been used to investigate tissue heterogeneity between synovial biopsies obtained from different patients in both juvenile RA [23] and long-standing RA [25,30]. Tsubaki and colleagues [23] used laser capture microdissection on biopsies retrieved by rheumatic arthroscopy from patients with juvenile RA to characterize proliferative lesions in the synovial lining. Two subgroups were discovered; one had a gene expression profile similar to that of long-standing RA. Van der Pouw Kraan and colleagues [25,30] used MA to investigate heterogeneity between synovial biopsies obtained by orthopedic surgery from different patients. In both of these studies, at least two different gene expression profiles were observed, which were suggested to correspond to high and low inflammatory status.

These and other studies therefore suggest that the MA technique might indeed be able to discern variable molecular features of the joint inflammation that would be both biologically and clinically meaningful. However, further investigation of the potential of these gene expression patterns to predict disease course as well as the response to various therapies is hampered by an incomplete knowledge of the natural variability of gene expression within the inflamed joints of single patients and between different patients with RA. In this study we therefore compared variation in gene expression patterns at the biopsy site, between different sites, and between patients. We used inflamed synovial tissues of patients with RA obtained during open surgery and during rheumatic arthroscopy, which were our methods of choice for synovial tissue retrieval.

## Materials and methods

### Patients

Thirteen patients, all fulfilling the American College of Rheumatology classification criteria for RA [1], were included in this study. Synovial tissues were taken from seven of these patients with erosive, end-stage disease during knee joint replacement surgery at the Department of Orthopedic Surgery, Karolinska University Hospital, Sweden. No further data on the characteristics of this subgroup of patients were available. Synovial tissue was obtained from the other six patients by rheumatic arthroscopy solely for research purposes. The clinical characteristics of these patients (five women and one man) are shown in Table 1. All six arthroscopic patients were recruited from the outpatient clinic of the Karolinska University Hospital Rheumatology Unit, and all except one (patient 13) had clinical arthritis with effusion in at least one knee joint at the time of the investigation. All patients except one (patient 11) were using the disease-modifying anti-rheumatic drug methotrexate, four in conjunction with low-dose corticosteroids, and all except one were using nonsteroidal anti-inflammatory drugs. Patient 13 had been taking methotrexate for two months; the others had been doing so for more than six months. Patients 10, 11 and 13 had erosive disease. The Ethical Committee at the Karolinska Institute approved the study protocol and all patients gave informed consent.

### Synovial tissue, sampling and handling

#### Orthopedic samples (patients 1 to 7)

Knee joint replacement surgery was performed in accordance with standard procedures, during which three synovial tissue specimens were obtained from random sites and immediately handled by research personnel. Each biopsy was visually inspected to minimize non-inflammatory synovial tissue contamination. Each orthopedic biopsy was then split into two parts, one of which was used for the MA experiment; the other was saved for histochemical analysis (except for biopsy 3 of patient 2 which was used only for MA). After dividing the biospies they were snap frozen (within two minutes) in precooled isopentane and stored at -80°C until further use, to ensure high RNA quality. For patients 1 to 3 each half of a biopsy that was to be used in the MA experiment was further divided into three parts, hereafter referred to as sub-biopsies. In total this resulted in 39 specimens from the orthopedic patients (nine sub-biopsies from patients 1 to 3 and three biopsies from each of patients 4 to 7). The average weight of the biopsies from patients 1 to 3 before division into three parts was 99 mg, yielding an average sub-biopsy weight of 33 mg. The average weight of the biopsies from patients 4 to 7 was 29 mg.

#### Arthroscopic samples (patients 8 to 13)

Rheumatic arthroscopy was performed by a technique previously described [31], including biopsy site-scoring for signs of inflammation (vascularity and proliferation), photography and mapping according to local standards (not shown). Biopsies were taken at the site of inflammation close to cartilage or not close to cartilage, defined as less than or more than 1.5 cm away from cartilage, respectively. Multiple biopsies were taken from two sites in patients 8, 11, 12 and 13 and from four sites in patients 9 and 10. The samples were frozen, as above, within two minutes. The average weight of the arthroscopic samples was 19 mg.

### Histochemistry

Frozen biopsies were embedded in Optimal Cutting Temperature (OCT; Tissue-Tek, SAKURA Finetek, Zoeterwoude, Netherlands) and cut with a cryostat into 7 μm thick. Sections were placed on SuperFrost^®^Plus slides (Menzel-Gläser, Braunschweig, Germany) and air-dried for 30 minutes, then stained with Mayer's hematoxylin and eosin to confirm the histopathology of each biopsy.

### RNA extraction

RNA was successfully extracted from all biopsies except biopsy 3 of patient 7, in which the RNA was degraded. For both types of biopsy (arthroscopic and orthopedic) one biopsy yielded enough RNA to perform MA experiments. To extract the RNA the biopsies were placed in steel-bead matrix tubes (Lysing Matrix D; Qbiogene, Irvine, CA, USA) containing buffer (600 μl of phenol, 600 μl RLT of buffer from an RNeasy kit (Qiagen, Hilden, Germany) and 0.6 μl of 2-mercaptoethanol) and homogenized with a tabletop FastPrep homogenizer (Qbiogene). The tubes were shaken for 30 seconds at speed setting 6 and then put on ice for 30 seconds. This procedure was repeated four times to ensure thorough homogenization. The tubes were then centrifuged for 5 minutes at 12,000 r.p.m. All steps up to this point were performed at 4°C. The water phase was collected and transferred to Qiashredder (Qiagen) columns and centrifuged (13,000 r.p.m. at room temperature) for two minutes to ensure complete homogenization. To the flow-through was added 600 μl of 70% ethanol.

The mixture was loaded onto RNeasy spin columns (Qiagen) and centrifuged for 15 seconds at 13,000 r.p.m. An RNeasy kit from Qiagen was used to wash and elute the extracted RNA (in 30 μl of RNase-free water). Before eluting the RNA the columns were treated with 2 units of DNAse H (Omega Biotech, Victoria, Canada) for 15 minutes at room temperature to remove residual DNA contamination. For further details see 'Preparation of RNA from tissues' at the KTH microarray core facility web site [32], under 'Protocols'. The average biopsy weight used for RNA extraction was 28.7 mg and the average RNA yield was 411.4 ng/μl in 30 μl. All concentration measurements were made with the Nanodrop (Nanodrop Technologies, Wilmington, DE USA). RNA quality was ensured with the RNA 6000 Nano LabChip kit of the Bioanalyzer system (Agilent Technologies, Palo Alto, CA, USA) where pass or fail judgments were based on an evaluation of Bioanalyzer electropherograms [33]. Two samples showed signs of partial degradation (patient 3, biopsy 3, and patient 6, biopsy 1). The average ratio of 28S to 18S rRNA among the remaining samples was 1.6.

### RNA amplification

Because of the small amounts of RNA extracted, the RNA was amplified with a RiboAmp RNA amplification kit (Arcturus, Mountain View, CA, USA). RiboAmp uses T7-based *in vitro *transcription to generate amplified RNA (aRNA), the bulk of which consists of sequences 250 to 1,800 base pairs long. Total RNA (300 ng to 1 μg) was used in each RNA amplification, and the average yield was 503 ng/μl in 11 μl of water.

### RNA reference

Universal Human Reference RNA from Stratagene (La Jolla, CA, USA) was used as reference RNA and was amplified in the same manner as the sample RNA. The reference RNA was pooled before use for hybridization.

### Labeling and cDNA synthesis

To prime the reaction, 1 μl of random hexamer primer (5 μg/μl; Operon, Alameda CA, USA) was added to 1 μg of amplified aRNA. The volume was adjusted to 18.4 μl with RNase-free water. The sample was mixed and incubated for 10 minutes at 70°C to denature the aRNA, then incubated for a further 5 minutes on ice and centrifuged briefly. A cDNA synthesis mixture (11.6 μl) consisting of 6 μl of 5× first-strand buffer, 3 μl of 0.1 M dithiothreitol, 2 μl of Superscript III (Invitrogen, San Diego, CA, USA) and 0.6 μl of 50× aa-dUTP+dNTP mix (Sigma-Aldrich, St. Louis, MO, USA) was added to each sample. The whole mixture was gently mixed by pipetting and incubated at 25°C. After 10 minutes at this temperature the mixture was incubated at 46°C for a further 2 hours. To terminate the reaction and to hydrolyze the RNA strand, 3 μl of 0.2 M EDTA pH 8.0 and 4.5 μl of 1 M NaOH were added. The sample was vortex-mixed briefly, incubated for 15 minutes at 70°C, cooled to room temperature and centrifuged briefly. Then 4.5 μl of 1 M HCl was added to restore the pH to neutrality. The sample was vortex-mixed and centrifuged briefly.

The cDNA was purified and eluted by the following procedure. First, 60 μl of water and 500 μl of PB buffer (MinElute Reaction Cleanup Kit; Qiagen) were added. The mixture was thoroughly mixed and transferred to a MinElute Reaction Cleanup Kit spin column and centrifuged for 30 seconds at 13,000 r.p.m. The flow-through was reapplied to the column and the centrifugation step was repeated. The flow-through was discarded and 650 μl of 80% ethanol was added to the column. The column was centrifuged for 30 seconds at 13,000 r.p.m. and the flow-through was again discarded. The ethanol wash step was repeated and then the membrane was dried by centrifugation for 1 minute at 13,000 r.p.m. The column was transferred to a new tube, and 10 μl of 100 mM NaHCO_3 _pH 9.0 was added. The column was then incubated for 1 minute at room temperature; the sample was eluted by centrifugation for 30 seconds at 13,000 r.p.m. The elution step was repeated after a further addition of 10 μl of 100 mM NaHCO_3 _pH 9.0 to ensure high yield.

To couple fluorophores, the eluate was mixed with a dried aliquot of either Cy3 or Cy5 mono-reactive esters (Amersham-Biosciences, Little Chalfont, Bucks., UK) and incubated for 30 minutes at room temperature in a dark container, after which 70 μl of water and 500 μl of PB buffer were added. The mixture was thoroughly mixed and transferred to a MinElute Reaction Cleanup Kit spin column, which was centrifuged for 30 seconds at 13,000 r.p.m. The flow-through was reapplied to the column and the centrifugation step was repeated. The flow-through was discarded and 650 μl of PE buffer (MinElute Reaction Cleanup Kit) was added. The column was centrifuged for 30 seconds at 13,000 r.p.m. and the flow-through was discarded. The wash step was repeated and then the membrane was washed by centrifugation for 1 minute at 13,000 r.p.m. The column was transferred to a new tube, 10 μl of EB buffer (MinElute Reaction Cleanup Kit) was added, and the column was incubated for 1 minute at room temperature. The sample was eluted by centrifugation for 30 seconds at 13,000 r.p.m. The elution step was repeated after a further addition of 10 μl of EB buffer, to ensure high yield. The concentrations of the incorporated fluorophore and cDNA were measured with the Nanodrop to confirm success in the labeling reaction. The sample was then ready for hybridization. For further information about the preparation of *N*-hydroxysuccinimide-ester fluorophores and indirect labeling of cDNA see SOP 001 and SOP 002 at the KTH microarray core facility web site [32] under 'Protocols'.

### cDNA microarray

The cDNA arrays used in this study were produced at the KTH microarray core facility. The clones on the array originate from the first 310 96-well plates of a commercial clone collection containing 46,000 sequence-verified human cDNA clones (Research Genetics; now Invitrogen). The clones have been prepared by cell culture, plasmid preparation, PCR amplification and purification with PCR filter plates (Millipore, Bedford MA, USA). The cDNA was spotted in 30% dimethylsulphoxide onto UltraGAPS slides (Corning, NY, USA) with a QArray spotter (Genetix, Hampshire, UK) with 24 SMP2.5 pins (Telechem, Sunnyvale, CA, USA) in 48 blocks, each of which contained 25 × 25 clones, spotted with a center-to-center distance of 170 μm, non-specifically attached to the surface by UV crosslinking. According to a UniGene mapping performed in September 2004 based on GenBank accession numbers (29,717 on the whole chip), 25,087 of the 30,000 spots on the chip have a UniGene ID and 16,164 of those were unique. For more information about the chip see the KTH microarray core facility web site [32] under 'HUM 30k cDNA array'.

### Hybridization

After prehybridizing of the slides for 30 minutes at 42°C in prehybridization buffer consisting of 1% BSA (Sigma-Aldrich), 5 × SSC (where SSC consists of 0.15 M NaCl and 0.015 M sodium citrate) and 0.1% SDS, the arrays were washed, first in a trough containing water and then in a trough containing propan-2-ol, and centrifuged dry. After that the samples (one labeled with Cy5 and the other labeled with Cy3) were pooled and dried to a volume of 13.6 μl. Hybridization buffer consisting of 5 × SSC, 50% formamide (Sigma-Aldrich), 0.1% SDS and 0.2 μg/μl Cot-1 DNA (Invitrogen) was added to the pooled samples to a final volume of 64.5 μl. The hybridization mixture was then denatured for 3 minutes at 95°C and cooled for 2 minutes on ice before being applied to the array. Lifter-slips (Erie Scientific Company, Shelton, CT, USA) were used to contain the hybridization mixture on the array during hybridization. The arrays were then placed, in hybridization chambers (Corning), in a water bath at 42°C for 14 to 18 hours. After hybridization the slides were washed once for 5 minutes at 42°C in wash buffer 1 (2 × SSC, 0.1% SDS), once for 5 minutes in wash buffer 2 (0.1 × SSC, 0.1% SDS) and five times, for 1 minute each, in wash buffer 3 (0.1 × SSC). The slides were then centrifuged dry and scanned. For further information about the hybridization see SOP 003 at the KTH microarray core facility web site [32] under 'Protocols'.

### Scanning and image processing

An Agilent G2565BA scanner was used to scan the slides and acquire 50-megabyte TIFF images. The scanner resolution was set at 10 μm. GenePix 5.1.0.0 (Axon Instruments, Foster City, CA, USA) was used to extract the raw signals from the TIFF images and to assign each spot an ID. Spots defined as 'not found' by GenePix were flagged with a negative flag (- 50) and removed downstream in the analysis. Spots with clearly abnormal morphology due to dust particles or other factors were manually flagged as bad (- 100) and were also removed in downstream analysis. No further processing of the slides was performed in GenePix. The data are available at ArrayExpress, a public repository for MA data (accession number E-MEXP-367) [34].

### Data analysis

The data were analyzed mainly with the help of packages in R [35] except for the Expression Analysis Systematic Explorer (EASE) analysis [36], which was performed in MEV [37]. EASE uses the hierarchical structure of gene ontology (GO) [38] to find biological themes among sets of differentially expressed (DE) genes. Each GO category is given an EASE score, which is a conservative adjustment of Fisher's exact probability [39] in which Fisher's exact probability is jackknifed to weigh significance in favor of GO categories supported by many genes. R is a language and environment for statistical computing and graphics. The packages that were used in R were LIMMA [40], aroma [41], the KTH package [32] and bioconductor [42]. All operations performed on the data in R during analysis can be accomplished with these packages. After the result files (gpr) produced by GenePix had been imported into R, unreliable spots with abnormal physical properties were removed with four filters:

1. filterFlags, which removes spots flagged as not found or absent in GenePix.

2. filterSaturated, which removes spots saturated in both cy5 and cy3.

3. filterB2SD, which removes spots in which 70% of the pixels have below background intensity + 2 standard deviations.

4. filterSize, which removes spots that are enlarged due to spotting artefacts.

On average 4,030 spots were removed by the filters, leaving about 25,970 spots for downstream analysis. More information about the filters can be found at the KTH package web site [32]. After filtering, the slides were normalized with print-tip loess (local regression) normalization [43]. To identify DE genes a parametric empirical Bayes approach implemented in LIMMA [40,44] was used. This test statistic will assign a score (*B*-score) to each gene. The *B*-score was used to rank the genes so that the gene with the highest score has the highest probability of being DE. When differences were being investigated, two criteria had to be fulfilled for a gene to be regarded as DE: the genes had to have a *B*-score of more than 0 and an |*M*-value| of more than 1 (an *M*-value is the second logarithm of the fold change [43]). When the DE genes were used to cluster the biopsies a third criterion was added: a gene had to occur in at least two or more comparisons between biopsies (regardless of patient) to be used for clustering. This was done to remove noise, because no biological replication was possible. When we were identifying DE genes between tissues with an overrepresentation of adipose cells versus all the other biopsies, we defined a gene as DE if the gene had a *B*-score of more than 20. Here samples from all patients were used, allowing the approximation of the different parameters used for the test statistics, for example the standard error, to be improved, and thus the *B*-scores to be high. A cutoff was therefore set at a *B*-score of more than 20 to investigate a reasonable number of genes with the highest ranking in this comparison.

A moderated *t *test [40] was performed in parallel, with the use of a false discovery rate [45] correction for multiple testing. Technical replicates were dealt with in different ways depending on the comparison in question. When three different levels of replicates were available (for example, for patients 1 to 3 the levels were technical replicates (i), multiple samples from each biopsy (sub-biopsies) (ii) and the biopsy (iii) itself), instead of just taking the average of technical replicates, we used the duplicateCorrelation function [46] available in LIMMA [40] to acquire an approximation of gene-by-gene variance. This retains valuable information about the variance when fitting a linear model to the data so as to identify DE genes. When four levels were available, as when testing for DE genes between patients 1 and 3 (technical replicates, sub-biopsies, biopsies and patients), the first level was averaged and duplicateCorrelation was used for the sub-biopsies and biopsies. When only two levels of replicates were available (for example when testing for DE genes between biopsies in patient 4 there were technical replicates of each biopsy that was tested) the replicates were all treated in the same way.

Several hierarchical clusterings were performed [47], in which 1 minus the Pearson correlation was used as the distance measure. When creating the agglomerative dendrogram the average distance between each cluster was used. To evaluate the structure the clustering algorithm imposes on the data the cophenetic (coph) correlation coefficient [48] was determined. This measures how well the hierarchical structure from the dendrogram represents the actual distances; coph = 1 indicates perfect representation, whereas coph = 0 indicates no representation. To facilitate color representation in the hierarchical clustering the (log_2_) expression value for each gene in each of the biopsies was adjusted by subtracting the respective mean log_2 _expression value across all biopsies.

### Experimental design

**Figure 1:**
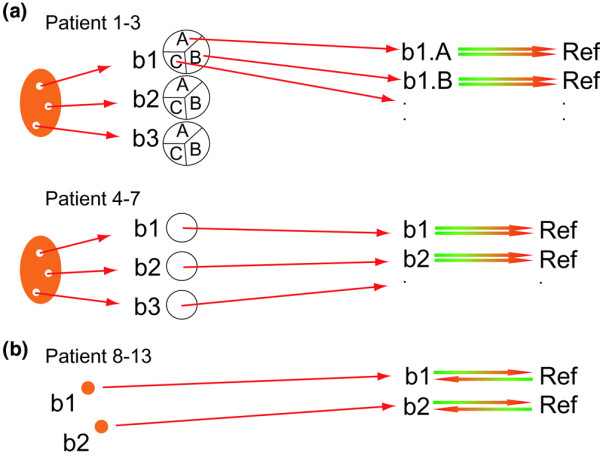
Experimental design. **(a) **Orthopedic samples. Three orthopedic biopsies (b1, b2, and b3) were taken from each of seven patients at random sites of the synovium. For patients 1 to 3 each biopsy was split into three parts. RNA from each sub-biopsy was hybridized in duplicate (b1.AA and b1.AB refer to biopsy 1, sub-biopsy A, technical replicates A and B, respectively) versus the reference. The average weight of the sub-biopsies was 33 mg. For patients 4 to 7, RNA from each biopsy was hybridized in duplicate (b1.A and b1.B: biopsy 1, technical replicates A and B, respectively) versus the reference. The average weight of the biopsies from patients 4 to 7 was 29 mg. **(b) **Arthroscopic biopsies. Two biopsies (b1 and b2) were taken from each of patients 8 and 11 to 13, and four from each of patients 9 and 10. Each biopsy was hybridized in duplicate versus the reference. The average weight of the arthroscopic biopsies was 19 mg.

Two series of hybridizations were performed in this study. The design chosen for both series was a common reference design that allows both the identification of DE genes in different contexts and unsupervised classification, such as hierarchical clustering. Each hybridization was performed with a technical replicate (the same amount of RNA taken from the same amplified RNA aliquot labeled in two separate reactions and hybridized onto two separate arrays). The average correlation between the *M*-values of technical replicates was 0.97. In the first series (Figure 1a), aRNA from the orthopedic sub-biopsies/biopsies (depending on the patient they were obtained from; see above) was labeled and hybridized in duplicate versus the reference (also amplified). In all, 76 hybridizations were performed in this series. In the second series (Figure 1b), aRNA from the arthroscopic biopsies was labeled and hybridized in duplicate versus the reference. A total of 32 hybridizations were performed in this series. The gene expression data were filtered and normalized as described above.

## Results

### Heterogeneity between orthopedic biopsies

**Figure 2:**
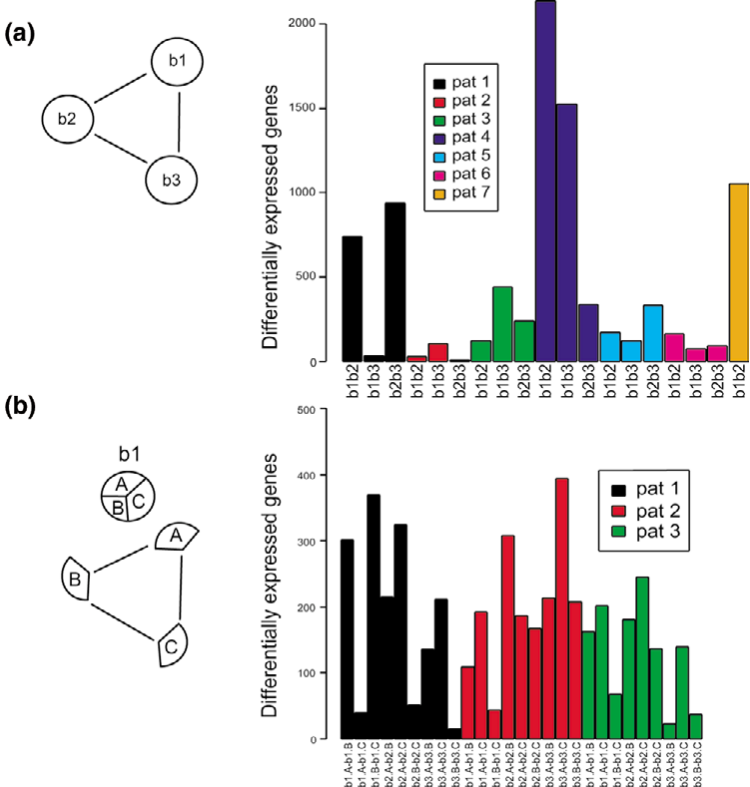
Differentially expressed genes between orthopedic biopsies and sub-biopsies. **(a) **As shown in the scheme at the left, comparisons were made patient by patient between all biopsies from all seven patients. Because of the degradation of RNA in the third biopsy, only two biopsies from patient 7 could be compared. The results from the between-biopsy comparisons can be seen in the barplot at the right. The first comparison to the left in the barplot b1b2 represents the comparison between biopsies 1 and 2 of patient 1. In total, 4,175 unique genes were found to vary between the different biopsies. **(b) **Comparisons were performed patient by patient and biopsy by biopsy between each sub-biopsy for patients 1, 2, and 3 in accordance with the scheme at the left. The results from all comparisons are displayed in the barplot at the right. The first comparison at the left in the barplot (b1.A-b1.B) illustrates the comparison between sub-biopsies A and B of biopsy 1 of patient 1. In total, 2,493 unique genes were found to vary between the sub-biopsies.

Two series of hybridizations were performed to investigate tissue heterogeneity, as indicated in Figure 1, covering variation both between orthopedic biopsies and between arthroscopic biopsies. In addition, the orthopedic biopsies of patients 1 to 3 were each divided into three parts (sub-biopsies) to study variation in gene expression between adjacent parts of the individual biopsies. First, an analysis of the heterogeneity between the orthopedic biopsies (b1, b2, and b3) from the same patients (Figure 2a) was performed. To measure heterogeneity we applied MA technology and estimated the differences between samples by determining the number of DE genes. Few, if any, genes should be DE between homogeneous tissues. As described in Materials and methods a gene was considered to be DE if it had a *B*-score of more than 0 and an |*M*-value| of more than 1 (that is, a more than twofold change). The *B*-score is derived from a statistical test (a parametric empirical Bayes approach, as described in Materials and methods) used to identify DE genes [44]. The higher the *B*-score, the more evidence there is of differential expression. The differences in gene expression seen in Figure 2a were caused by both variation in cellular composition and true changes in gene expression due to various factors. In all, 2,133 genes were identified as DE when comparing biopsies 1 and 2 from patient 4. In contrast, only six genes were found to be DE when comparing biopsies 2 and 3 of patient 2. This demonstrates that there was a high level of variation between biopsies originating from the same joint, indicating that there may be large differences in cellular composition between synovial biopsies obtained from patients with RA.

### Heterogeneity between sub-biopsies

**Figure 3:**
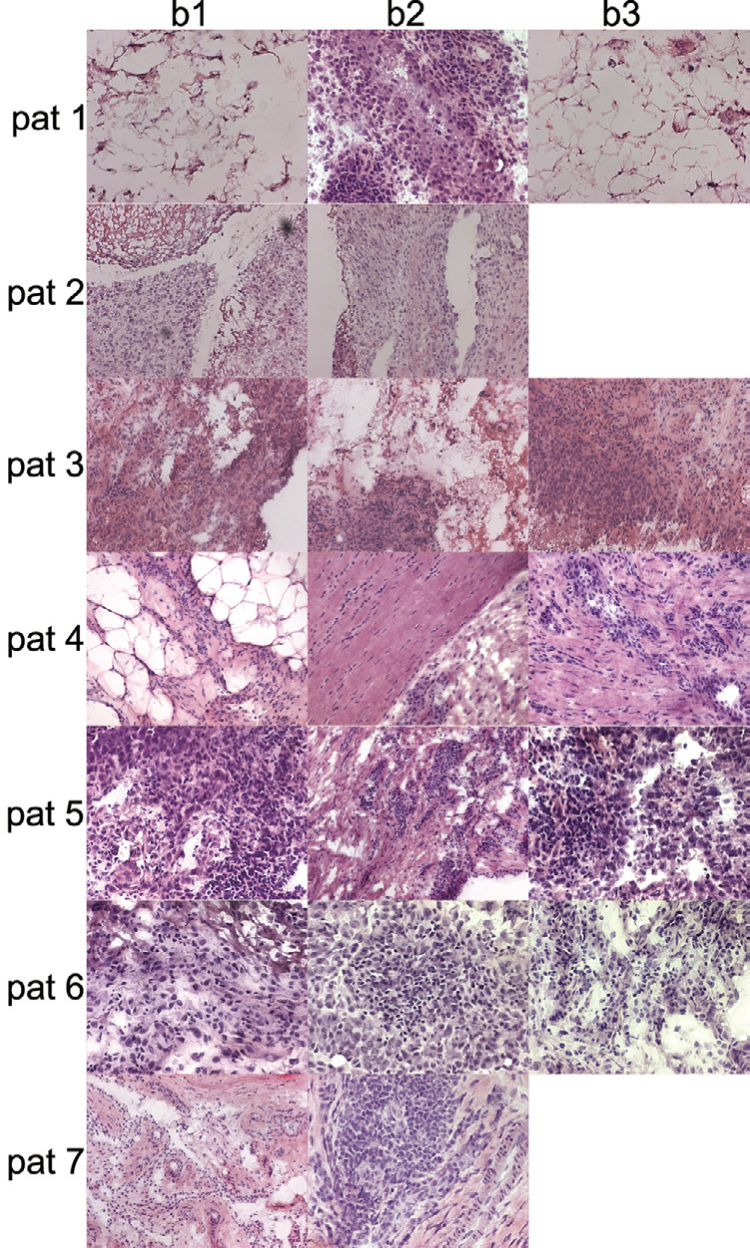
Hematoxylin/eosin staining of the synovial membrane of patients 1 to 7. Original magnification × 250. Biopsy 3 (b3) of patient 2 was available only for RNA extraction and not for staining. Biopsy 3 of patient 7 was not represented in the stainings or microarray analysis because of poor RNA quality. Biopsies 1 and 4 of patient 1, and biopsy 1 of patient 4, consisted mostly of adipose cells with some element of inflammatory cells. Biopsy 1 of patient 7 had a higher level of heterogeneity, and contained more vessels, than the others.

Variation between adjacent biopsies was investigated by separately analyzing gene expression in each of the three sub-biopsies derived from the biopsies obtained from patients 1 to 3 (Figure 2b). Interestingly, many genes were DE (*B*-score>0 and an |*M*-value|>1) between sub-biopsies, despite apparent homogeneity between biopsies, as exemplified by the biopsies of patient 2 (Figure 2a). However, overall the sub-biopsies were quite similar, with the average number of DE genes being 180, 202, and 132 for patients 1, 2, and 3, respectively, although significant differences were found in these comparisons. For example, biopsies b1 and b3 from patient 1 seemed to be quite similar (Figure 2a), but further investigation found great variation between sub-biopsies of biopsy b1 (Figure 2b). Here, the sub-biopsies A and C seemed similar but there seemed to be large variations in cellular composition between sub-biopsies A and B. Furthermore, the hematoxylin/eosin staining patterns demonstrated that biopsies b1 and b3 of patient 1 contained an overrepresentation of adipose cells (Figure 3), whereas b2 seemed to represent synovial hyperplasia. It should be noted that the differences between biopsies seemed to be larger than those between sub-biopsies.

### Hierarchical cluster of orthopedic biopsies

**Figure 4:**
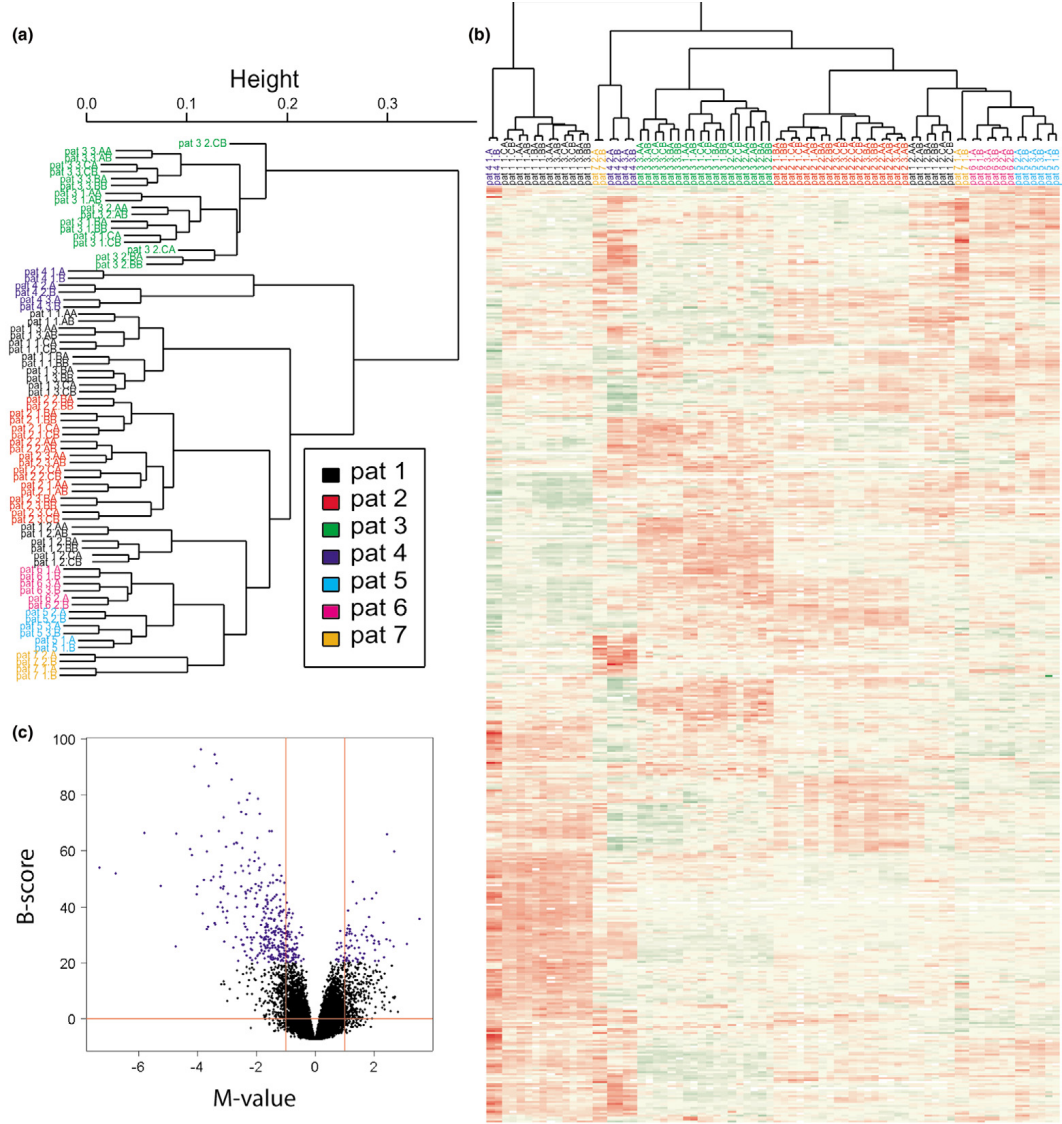
Hybridizations of orthopedic samples (patients 1 to 7). **(a) **Results of hierarchical clustering (HCL) performed on the raw data after normalization. On average, 26,111 features were used per array. Cophenetic (coph) correlation coefficient = 0.9. **(b) **HCL analysis of the 2,285 genes that were found to vary between biopsies and were present in one or more comparisons; coph = 0.92. Color codes: red, upregulated genes; green, downregulated genes; yellow, genes showing no change; white, missing values. For patients 1 to 3, the coding of samples in (a) and (b) is as follows: Pt2 1.BA refers to patient 2, biopsy 1, sub-biopsy B, technical replicate A. For patients 4 to 7, the coding of samples in (a) is as follows: Pt6 2.A refers to patient 6, biopsy 2, technical replicate A. **(c) **Volcano plot of the comparison between the adipose-enriched samples versus all other biopsies. Biopsies 1 and 3 of patient 1, and biopsy 1 of patient 4, were tested for differentially expressed genes against all other orthopedic biopsies. Genes with a *B*-score of more than 20 are shown in blue. Genes with highly significant differences in expression levels that were upregulated in the samples with an overrepresentation of adipose cells in comparison with other biopsies can be seen in the upper left corner.

Four levels of replicates were used in this study: technical replicates (the same amount of RNA taken from the same amplified RNA aliquot labeled in two separate reactions and hybridized onto two separate arrays); adjacent biopsies in the form of sub-biopsies; multiple biopsies obtained from different locations in the same joint and patient; and biological replicates in the form of biopsies from different patients. Hierarchical clustering (HCL) analyses (Figure 4) were initially performed to obtain an overview of the differences between the technical replicates, resampled biological replicates (sub-biopsies/biopsies), and biological replicates (patients). The first HCL analysis was performed (Figure 4a) on the raw data after filtration and normalization. As can be seen in the resulting clustering, the biopsies separate patient by patient except for those from patient 1. This is consistent with data presented in Figure 2a for this sample.

### Hierarchical cluster of orthopedic biopsies using a subset of genes

The DE genes that were found to vary between biopsies within patients (4,175 genes; Figure 2a) and were present in one or more comparisons (2,285 genes) were used to cluster the data (Figure 4b). As shown in the dendrogram, this subset of genes contains information that distinguishes between three biopsies (biopsies 1 of patient 1, biopsies 3 of patient 1, and biopsy 1 of patient 4). Overall the results of the HCL analysis in Figure 4b correlated well with the hematoxylin/eosin staining patterns (Figure 3), which demonstrated that these three biopsies had a clear overrepresentation of adipose cells relative to the other biopsies. Note that biopsies 1 and 2 of patient 7 also clustered separately, which is again explained by the histological data although the difference was visually not as obvious. The remaining biopsies clustered according to patient. The three biopsies with an overrepresentation of adipose cells were further investigated by determining the DE genes between these biopsies and all other biopsies. The results can be seen in Figure 4c. Genes with a *B*-score of more than 20 were chosen for further investigation as described in Materials and methods. False discovery rate [45] correction for multiple testing was applied on a moderated *T*-test, yielding a proportion of 1.03 × 10^-11 ^false positives among the 418 chosen genes with a *B*-score of more than 20, 341 of which were upregulated in the biopsies with an overrepresentation of adipose cells relative to the rest. These genes were used to elucidate possible biological themes with the use of the GO tool EASE [36]. The results of the EASE analysis (Table 2) show that the overrepresentation of adipocytes detected in the staining analysis is confirmed by the gene expression data.

### Heterogeneity between arthroscopic biopsies

**Figure 5:**
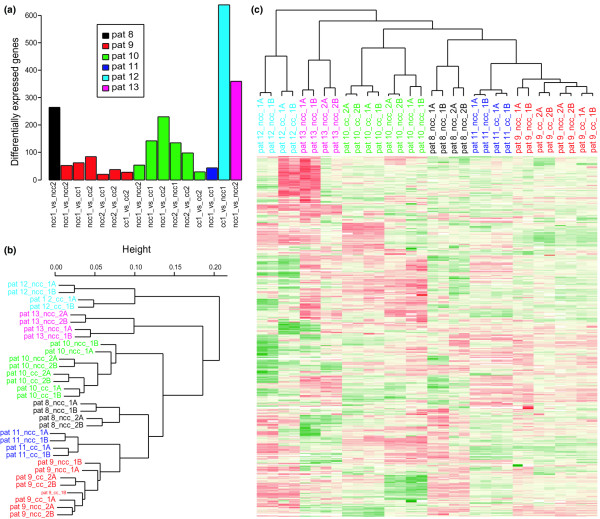
Hybridizations of arthroscopic samples (patients 8 to 13). **(a) **Barplot of the differentially expressed genes identified in the patient-by-patient comparisons between biopsies. In total, 1,536 unique genes were found to vary between the biopsies. **(b) **Hierarchical clustering (HCL) of the arthroscopic biopsies using all genes. Pt9_cc_2A, for example, refers to patient 9, distance to cartilage (ncc, not close to cartilage, cc, close to cartilage), biopsy 2, technical replicate A. Color codes: red, upregulated genes; green, downregulated genes; yellow, genes showing no change; white, missing values. (**c) **HCL analysis based on the subset of differentially expressed genes from (a) that was present in one or more comparison (421 genes).

In the second series of hybridizations (Figure 1b), comparisons were made between all of the arthroscopic biopsies from the respective patients. The barplot in Figure 5a shows the results of these comparisons. The largest difference (638 DE genes) was observed in patient 12 and the smallest between two biopsies from patient 9 (21 DE genes). The arthroscopic biopsies were clustered by using all of the features on the chip and also a subset of genes that were defined as DE in Figure 5a (1,536 genes) and were present in one or more comparisons (421 genes). The results of the cluster analysis can be seen in Figure 5b,c. In contrast to the orthopedic samples, the biopsies from the different patients clustered individually with both approaches.

### Differences between biopsies and patients

**Figure 6:**
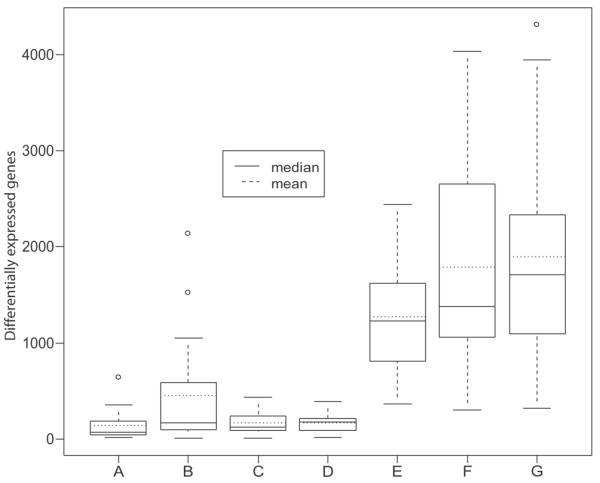
Boxplots of the differentially expressed (DE) genes in the different comparisons. Boxplot A shows the number of DE genes between the arthroscopic biopsies shown in Figure 5a. Boxplot B shows the number of DE genes between the orthopedic biopsies shown in Figure 2a. Boxplot C shows the number of DE genes between orthopedic biopsies excluding the comparisons involving biopsies 1 and 3 of patient 1, biopsy 1 of patient 4, and biopsy 1 of patient 7. Boxplot D shows the number of DE genes between the sub-biopsies as shown in Figure 2b. Boxplot E shows the number of DE genes between the arthroscopic patients. Boxplot F shows the number of DE genes between the orthopedic patients. Boxplot G shows the number of DE genes between the orthopedic patients excluding biopsies 1 and 3 of patient 1, biopsy 1 of patient 4, and biopsy 1 of patient 7 from the comparisons.

The average number of DE genes was 143 between the arthroscopic biopsies (Figure 5a) and 455 between the orthopedic biopsies (Figure 2a), indicating that there was less heterogeneity among the arthroscopic biopsies than among the orthopedic samples. The distribution of differences between orthopedic and arthroscopic biopsies is illustrated in boxplots A and B of Figure 6, respectively. As can also be seen in boxplot C of Figure 6, when the biopsies containing an overrepresentation of adipose cells are removed from the orthopedic biopsies the distribution of DE genes becomes very similar to that of the arthroscopic biopsies, reducing the average number of DE genes between biopsies from 455 to 171. Interestingly, the heterogeneity between sub-biopsies (boxplot D of Figure 6; 173 DE genes on average) is similar to that of the reduced set of orthopedic biopsies. This indicates that the gene expression heterogeneity between adjacent biopsies is similar to that of biopsies farther apart if the biopsies consist of the same type of tissue. In addition, we compared sub-biopsy/biopsy variation with patient variation. As with the comparisons between sub-biopsies/biopsies, a gene was regarded as being DE if it had a *B*-score of more than 0 and an |*M*-value| of more than 1. The results are displayed in Figure 6, boxplots E to G. The same patterns that were observed between biopsies were also seen between patients, because the orthopedic patients were more heterogeneous than the arthroscopic patients. However, the differences were reduced when the biopsies that were not suitable for gene expression analysis of synovial inflammation were removed. EASE [36] was used to investigate the possible overrepresentation of biological processes by sorting the genes found to vary between biopsies into subsets based on GO categories. The numbers of 'hits' and the EASE scores for the 10 most overrepresented GO categories are shown in Table 3 for both the orthopedic (4,175 unique genes) and the arthroscopic (1,536 unique genes) biopsies. No major differences in the relative representation of biological processes between the two types of samples were found in this analysis.

## Discussion

The purpose of this study was to investigate variations in gene expression in synovial tissues, within and between patients with RA, using MA technology. The study also allowed the sampling techniques used (orthopedic open surgery and rheumatic arthroscopy) to be compared. The results show large differences in the numbers of DE genes between biopsies, supporting previous studies on tissue heterogeneity in synovial joints affected by inflammatory disease [25,30]. The results also demonstrate that the differences between patients are larger than between biopsies obtained from the same joint (Figure 6). For the arthroscopic biopsies the unique gene expression signature of each patient dominated the dendrograms obtained using both all genes and the subset of DE genes. This was not observed for the orthopedic biopsies. Most of the orthopedic biopsies clustered according to the patient, but some failed. Instead, they clustered according to their cellular composition; that is, mainly in relation to their content of adipose cells as detected by parallel histology. This has potential implications for how MA analysis can be used to classify and follow the course of patients with arthritis, and in particular for choosing the optimal sampling technique.

Rheumatological arthroscopy has the advantage that the investigator can choose a section of the synovium that is inflamed (vascular and proliferative changes) and avoid much fat or fibrous tissue. In contrast, orthopedic biopsies are most often taken during operation in an extremity where blood supply has been temporarily turned off and where it is difficult to separate inflamed from fibrous or fatty tissue at macroscopic sampling of biopsies. The increased precision in biopsy sampling from inflamed areas during arthroscopic compared with sampling during open joint surgery might therefore explain the patient-restricted clustering for arthroscopic but not for orthopedic biopsies. This observation also has implications for the choice between the use of arthroscopic or blind-needle biopsies, in which sampling of biopsies under direct vision during arthroscopy is more likely to focus on areas of inflammation than sampling with the blind-needle biopsy technique. In addition, the orthopedic samples were taken from patients undergoing end-stage arthroplastic surgery, whereas the samples taken during arthroscopy were from patients with a much shorter disease duration (2.3 years). When samples containing substantial fat tissue rather than inflammatory cells were removed from the analysis, biopsies clustered almost perfectly both at the level of biopsy site and individually.

It therefore seems that biopsies from actively inflamed synovial tissues of patients with RA show expression of unique patterns of mRNA, provided that the biopsy has been taken in such a way that the analysis is performed on cells from an inflamed site. Thus, both inter-individual and intra-individual variation must be taken into consideration when analysing gene expression in synovial tissue. One way is to take multiple biopsies, as suggested earlier [8]; however, this would reduce gene expression changes due to local differences in gene expression at each biopsy site. Instead we suggest taking one biopsy from a site of maximal macroscopic inflammation. If reproduced in still larger series of biopsy studies using the current as well as additional array methodologies, it is therefore feasible that the MA technology performed on single biopsies might provide information that allows us to investigate the value of this information in predicting disease course as well as response to therapy, and to follow the results of therapies over the longitudinal course of a chronic inflammatory disease such as RA.

## Conclusion

The results in this paper demonstrate that levels of homogeneity vary between synovial biopsies obtained from the same knee. Nevertheless, the gene expression signature of each biopsy was patient-specific except for four orthopedic biopsies. These were identified by gene expression analysis and confirmed by histochemistry as not being suitable for the analysis of synovial inflammation because of tissue heterogeneity. The results also demonstrate differences between two different sampling methods: open surgery and arthroscopy. The number of DE genes between the orthopedic biopsies was on average almost threefold higher than in the arthroscopic biopsies.

## Abbreviations

aRNA = amplified RNA; coph = cophenetic correlation coefficient; DE = differentially expressed; EASE = Expression Analysis Systematic Explorer; GO = gene ontology; HCL = hierarchical clustering; MA = microarray; PCR = polymerase chain reaction; RA = rheumatoid arthritis.

## Competing interests

The authors declare that they have no competing interests.

## Authors' contributions

J Lindberg performed parts of the MA-related laboratory work, contributed to the data analysis, and wrote parts of the article. EaK performed the arthroscopic surgery, wrote parts of the article, and contributed to the data analysis. A-KU contributed to the planning and design of the project, collected the orthopedic biopsies outside surgery, performed the hematoxylin/eosin staining analysis, and participated in both the data analysis and writing of the article. AS performed the joint replacement surgery and thereby provided the orthopedic biopsies. TA contributed to the planning and design of the project and performed parts of the MA-related laboratory work. PN was responsible for chip design and contributed to data analysis. LK was involved in planning the project, data analysis, and writing the article. J Lundeberg was involved in designing the project, the analysis of laboratory results, data analysis, and writing of the article. All authors read and approved the final manuscript.

## Figures and Tables

**Table 1:** Clinical data for patients 8 to 13

Patient	Diagnosis	RF	Sex	Age (years)	Age at diagnosis	Duration of arthritis (weeks)	DMARD	Corticosteroids	NSAID
8	RA	-	F	57	4 years	2	Methotrexate 10 mg/week	No	Ketoprofen 200 mg/day
9	RA	-	F	69	6 months	52	Methotrexate 10 mg/week	Prednisolone 7.5 mg/day	No
10	RA	+	F	52	4 years	1	Methotrexate 17.5 mg/week s.c.	Prednisolone 5 mg/day	Ketoprofen 200 mg/day
11	RA	+	F	64	4 days	3	No	Prednisolone 7.5 mg/day since 4 days	Diklofenac 150 mg/day
12	RA	+	F	57	9 months	26	Methotrexate 7.5 mg/week	Prednisolone 7.5 mg/day	Indometacin 75 mg/day
13	RA	+	M	66	10 weeks	n.a.	Methotrexate 10 mg/week	No	Diclofenac 50 mg on demand

DMARD, disease-modifying anti-rheumatic drug; NSAID, non-steroidal anti-inflammatory drug; RA, rheumatoid arthritis; RF, rheumatoid factor serology; s.c., subcutaneous.

**Table 2:** EASE results

GO ID	GO term	Number of hits	EASE score
GO:0006629	Lipid metabolism	24	6.17 × 10^-6^
GO:0006631	Fatty acid metabolism	10	1.67 × 10^-4^
GO:0006091	Energy pathways	13	1.71 × 10^-4^
GO:0019752	Carboxylic acid metabolism	16	7.72 × 10^-4^
GO:0006082	Organic acid metabolism	16	8.29 × 10^-4^

The table lists Expression Analysis Systematic Explorer (EASE) results on the 341 genes upregulated in the biopsies with an overrepresentation of adipose cells versus the rest of the orthopedic biopsies. The five most significant gene ontology (GO) categories are shown.

**Table 3:** EASE results for differentially expressed genes identified in hybridization series 1 and 2

GO ID	GO term	Orthopedic biopsies			Arthroscopic biopsies		
		Rank	Number of hits	EASE score	Rank	Number of hits	EASE score
GO:0007154	Cell communication	1	685	4.63 × 10^-14^	5	273	4.72 × 10^-6^
GO:0007155	Cell adhesion	2	166	3.29 × 10^-10^	7	72	8.51 × 10^-6^
GO:0009607	Response to biotic stimulus	4	199	5.89 × 10^-9^	3	90	1.30 × 10^-6^
GO:0006952	Defense response	5	181	1.60 × 10^-8^	2	84	7.69 × 10^-7^
GO:0009605	Response to external stimulus	6	273	3.00 × 10^-8^	1	126	1.56 × 10^-7^
GO:0006955	Immune response	7	166	1.12 × 10^-7^	4	78	1.63 × 10^-6^
GO:0007166	Cell surface receptor linked signal transduction	9	209	1.24 × 10^-6^	9	95	1.77 × 10^-5^
GO:0007160	Cell-matrix adhesion	10	31	2.98 × 10^-5^	121	9	2.32 × 10^-1^
GO:0009613	Response to pest/pathogen/parasite	11	107	3.66 × 10^-5^	6	55	6.78 × 10^-6^
GO:0009611	Response to wounding	21	61	1.43 × 10^-3^	8	36	1.44 × 10^-5^
GO:0006954	Inflammatory response	53	40	1.89 × 10^-2^	10	27	3.77 × 10^-5^
GO:0007165	Signal transduction	3	533	4.69 × 10^-10^	20	204	1.77 × 10^-3^
GO:0009987	Cellular process	8	1,303	1.95 × 10^-7^	15	519	4.28 × 10^-4^

The table lists the top 10 gene ontology (GO) categories for the 4,175 genes that were differentially expressed between orthopedic biopsies and for the 1,536 differentially expressed genes that were found to vary between arthroscopic biopsies. Seven of the top 10 GO categories were common between the two biopsy types. In the columns headed 'Rank', category 1 is the most significant category and the category with the highest number is the least significant. EASE, Expression Analysis Systematic Explorer.
